# Reduction of UreB and CagA expression level by siRNA construct in *Helicobacter pylori* strain SS1

**DOI:** 10.1186/s12866-023-03143-x

**Published:** 2023-12-19

**Authors:** Hamid Motamedi, Ramin Abiri, Farhad Salari, Cyrus Jalili, Amirhoushang Alvandi

**Affiliations:** 1 Department of Microbiology, School of Medicine, Kermanshah University of Medical Sciences, Kermanshah, Iran; 2 Student Research Committee, School of Medicine, Kermanshah University of Medical Sciences, Kermanshah, Iran; 3 Fertility and Infertility Research Center, Health Technology Institute, Kermanshah University of Medical Sciences, Kermanshah, Iran; 4 Department of Immunology, School of Medicine, Kermanshah University of Medical Sciences, Kermanshah, Iran; 5 Medical Biology Research Center, Health Technology Institute, Kermanshah University of Medical Sciences, Kermanshah, Iran; 6 Medical Technology Research Center, Health Technology Institute, Kermanshah University of Medical Sciences, Kermanshah, Iran

**Keywords:** ureB, cagA, siRNA, *Helicobacter pylori*

## Abstract

**Background:**

Two important virulence factors, urease and cagA, play an important role in *Helicobacter pylori* (*H. pylori*) gastric cancer. Aim of this study was to investigate the expression level and function of *ureB* and *cagA* using small interfering RNAs (siRNA).

**Methods:**

SS1 strain of *H. pylori* was considered as host for natural transformation. siRNA designed for *ureB* and *cagA* genes were inserted in pGPU6/GFP/Neo siRNA plasmid vector to evaluate using phenotypic and genotypic approaches. Then, qPCR was performed for determining inhibition rate of *ureB* and *cagA* gene expression.

**Results:**

The expression levels of siRNA-ureB and siRNA-cagA in the recombinant strain SS1 were reduced by about 5000 and 1000 fold, respectively, compared to the native *H. pylori* strain SS1. Also, preliminary evaluation of siRNA-ureB in vitro showed inhibition of urea enzyme activity. These data suggest that siRNA may be a powerful new tool for gene silencing in vitro, and for the development of RNAi-based anti-*H. pylori* therapies.

**Conclusion:**

Our results show that targeting *ureB* and *cagA* genes with siRNA seems to be a new strategy to inhibit urease enzyme activity, reduce inflammation and colonization rate.

## Introduction

*Helicobacter pylori (H. pylori)* is a Gram-negative microaerophilic bacterium that colonizes the gastric mucosa of half of the world's population and causes various diseases, including peptic ulcer disease (PUD), gastric mucosa-associated lymphoid tissue (MALT) lymphoma, and gastric cancer [1]. The prevalence of PUD in people with *H. pylori* infection is estimated at 10% [2]. According to the World Health Organization (WHO), *H. pylori* is classified as a class I carcinogen for causing gastric cancer [3]. Gastric cancer is one of the major health challenges worldwide, accounting for approximately 754,000 deaths annually, and is the fourth leading cause of cancer-related deaths in both sexes [4]. Among the virulence factors of *H. pylori* that are important in the pathogenesis of stomach disease are urease, cytotoxin-associated gene A (CagA), Vacuolating cytotoxin A (VacA) and adhesion proteins [5]. Phenotypic and genotypic methods can be useful in the diagnosis of H. pylori. So that in phenotypic methods such as culture and antibiotic sensitivity testing, it may be a prerequisite for patients with persistent infection after initial or repeated treatment failure [6], and on the other hand, molecular methods provide the possibility of rapid diagnosis of this bacterium as well as determination of its genotype [7].

Urease plays an essential role in the pathogenesis of *H. pylori* and constitutes 10–15% of the total bacterium protein by weight. In 2001, it was shown that *H. pylori* urease consists of two structural proteins, α (61.7 kDa) and β (approximately 26.5 kDa) subunits, which are located in the outer membrane of the bacterium [8]. Successful colonization by this bacterium in the acidic condition of the stomach requires active urease, which hydrolyzes urea to produce ammonia and CO_2_, allowing *H. pylori* to colonize the gastric mucosa [9]. On the other hand, *H. pylori* induces cellular and humoral immune responses of the host to the site of infection, and this immune response provides nutrients for the stomach pathogen and ultimately enables continuous colonization of the stomach throughout the host's life [10, 11]. Various compounds have been used to inhibit *H. pylori* urease enzyme [12, 13]. UreB subunit is the most effective and common immunogen of *H. pylori* that can create a protective immune response in the body against this bacterium, so UreB is a very good reporter of in vivo gene expression [14]. Therefore, urease activity and stability are necessary for colonization by *H. pylori* in the human stomach.

CagA protein is a 128–145 kDa protein encoded by the cag pathogenicity island (cagPAI), which can be injected into gastric epithelial cells via the type IV secretion system (T4SS) [15, 16]. CagA protein directly targets the gastric epithelium and causes CagA-mediated carcinogenesis, while VacA protein promotes apoptosis and epithelial cell death [17]. Evidence has shown that CagA causes acute gastritis, peptic ulcer, and the development of gastric and VacA toxin increases the ability of bacteria to colonize the stomach and contributes to the pathogenesis of gastric adenocarcinoma and peptic ulcer disease [18–20]. In a prospective study, the risk of duodenal ulcer and gastric ulcer was increased by 18.4-fold and 2.9-fold in individuals infected with cagA-positive *H. pylori* strains, respectively [21]. CagA can affect the host cell in several different ways: 1) changing host signaling in both phosphorylation-dependent and independent ways (e.g.: phosphorylated CagA binds to SHP-2 phosphatase and affects cell adhesion, proliferation and migration 2) altering the cytoskeleton, 3) affecting cell proliferation, and 4) stimulating gastric epithelial cells to secrete interleukin-8 (IL-8) [22, 23]. IL-8 is a major neutrophil-activating cytokine and also one of the most important chemokines for *H. pylori*-induced chronic gastric inflammation [24].

**Fig. 1:**
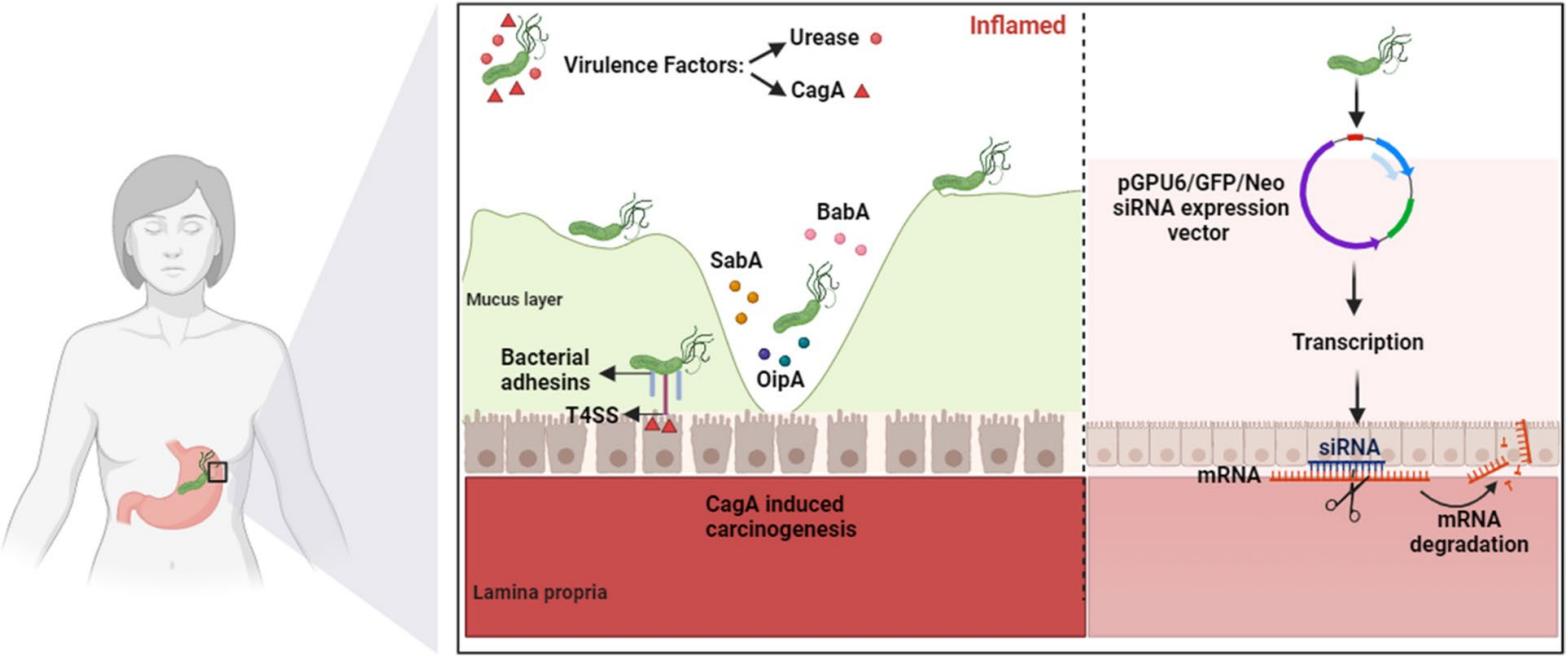
Effect of siRNA on Urease and CagA virulence factors of *H. pylori*. Urease is a critical factor that facilitates bacterial colonization in the gastric mucosa and hydrolyzes urea to produce ammonia and CO_2_. Proteins such as blood group antigen binding adhesin A (BabA), sialic acid–binding adhesin (SabA), and outer inflammatory protein A (OipA) produced by this pathogen contribute to colonization and persistence of infection. CagA protein directly targets gastric epithelium and CagA-mediated carcinogenesis occurs. In the siRNA pathway, full complementary binding between the siRNA guide strand and the target mRNA leads to mRNA cleavage. In other words, siRNA reduces or inhibits the expression level of *ureB* and *cagA* genes, which results in the reduction of colonization and inflammation. The figure is created using BioRender.com

Three decades ago researchers showed that small interfering RNAs (siRNAs) are double-stranded RNAs of 21–25 nucleotides that act as key effector molecules in triggering sequence-specific RNA degradation during post-transcriptional gene silencing [25]. Based on scientific evidence, siRNA molecules have been considered as potentially effective therapeutic targets for a wide range of diseases, including cancer, viral and bacterial infections [26, 27]. Meanwhile, five siRNA-based drugs (patisiran, givosiran, incilisiran, lumasiran, and veterisiran) have been approved by the Food and Drug Administration (FDA) and several powerful drugs are in the final stages of phase III clinical trials [28]. However, no study has been conducted on the inhibition of ureB and cagA virulence factors of *H. pylori* by siRNA. In Fig. 1, shows the effect of siRNA on urease and CagA virulence factors of *H. pylori*.

The aim of this study was to investigate the expression level and function of virulence factor *ureB* and *cagA* of *H. pylori* in the presence and absence of siRNA, which can be promising for the treatment of *H. pylori* infection and other diseases related to this bacterium.

## Methods

### Bacterial strain and growth condition

**Fig. 2:**
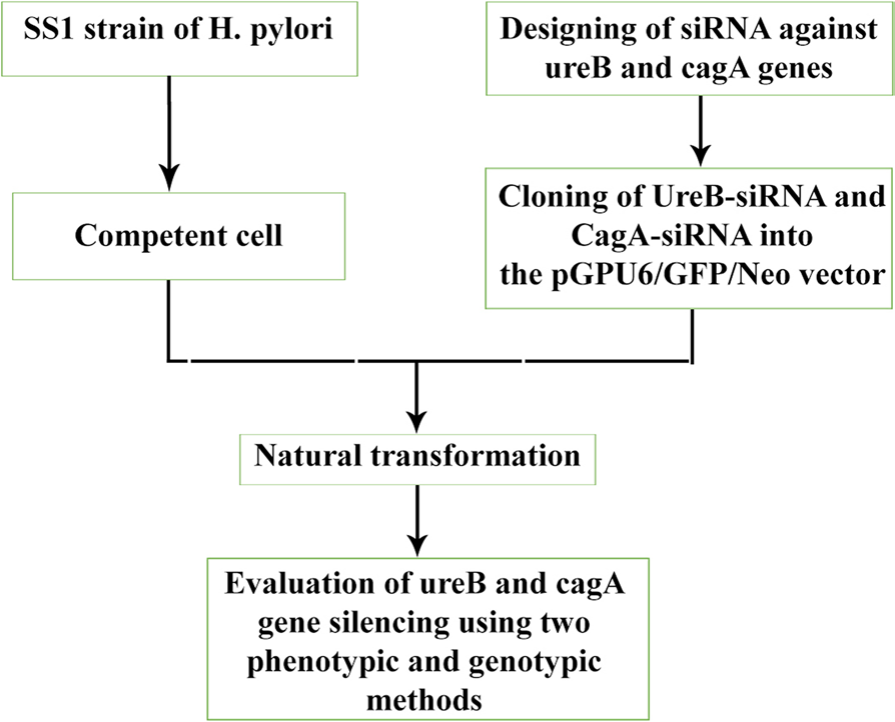
Flow chart of study design

The SS1 *H. pylori* strain was kindly provided by Dr. Mohammad Ali Haghighi (Bushehr University of medical sciences, Iran). The strain was cultured on Luria–Bertani agar (LB) medium (Difco Laboratories, USA, Detroit, MI) supplemented with 8% fetal calf serum (FCS), 5 μl/ml trimethoprim (Sigma), 5 μl/ml vancomycin, 2.5 units/ml polymyxin B (Sigma) and 8 μl/ml amphotericin B (Sigma). The microaerophilic conditions (85% N_2_, 10% CO_2_, 5% O_2_) was prepared using Anoxomat system (Advanced Instruments, Inc., Norwood, USA, MA) and were incubated for 3–4 days at 37 °C. The workflow for this scientific study is shown in Fig. 2.

### Obtaining small interfering RNAs

siRNA sequences against *ureB* and *cagA* genes were designed by siDirect 2.0 web server (http://siDirect2.RNAi.jp/), and after screening and BLAST (Basic Local Alignment Search Tool) analysis, the best siRNA was selected. This web server adjusted the parameters so that the siRNA candidates met the Ui-Tei, Reynolds and Amarzguioui (URA) criteria for improving specificity in in vitro assays. The max Tm value for seed target duplex stability was kept at 21.5 °C to reduce off-target effect [29]. Because the GC content of the siRNA duplex is related to its function, a range of 30% to 50% was determined via OligoCalc (http://biotools.nubic.northwestern.edu/OligoCalc.html). In addition, structural and thermodynamic analysis was performed using RNA structure (https://rna.urmc.rochester.edu/RNAstructure.html) to predict secondary structures in terms of their free bending energy at 37 °C. Finally, the effectiveness of siRNA inhibition was evaluated through the siRNApred web server (https://webs.iiitd.edu.in/raghava/sirnapred).

siRNA and pGPU6/GFP/Neo siRNA expression vector were synthesized by Shanghai GenePharma Co., Ltd. (Shanghai, China) and stored at -20 °C until use. The length of siRNA for each target gene was 21 base pairs. The target sequences were for siRNA-UreB-1352 5'- AAGGTGGGTTCATTGCATTAA-3' and siRNA-CagA-1656 5'- AGGCGGAATTTAGAGGATAAA-3'.

pGPU6/GFP/Neo siRNA expression vector has multiple insertion site for adding selected sequences and also has prokaryotic and eukaryotic markers (antibiotic resistance) and reporters for screening of recombinant host. This vector contains multiple promoters for siRNA expression in multiple hosts and a green fluorescent protein (GFP) expression cassette that can also be coexpressed with siRNAs.

### In silico cloning

The pGPU6/GFP/Neo plasmid constructs model were designed using SnapGene software (version 5.2.3) (https://www.snapgene.com/). Each constructs model was in silico analyzed for specific restriction enzyme pattern using SnapGene software.

### Natural transformation

**Fig. 3:**
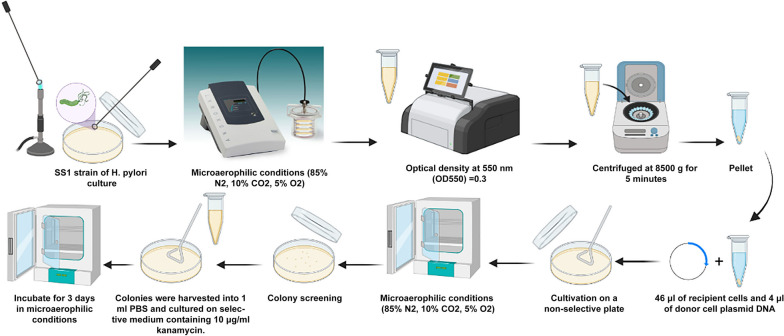
The stages of natural transformation. The figure is created using BioRender.com

Transformations were performed as previously described [30]. *H. pylori* strain SS1, which had grown for 3–4 days on LB plates with 8% FCS, was suspended in 1 ml of phosphate buffered saline (PBS; pH 7.4) (Fig. 3). When the optical density of the suspension at 550 nm (OD_550_) reached 0.3, it was centrifuged at 8500 g for 5 min and the pellet was resuspended in 150 μl of PBS. Each transformation mixture, consisting of 46 μl of recipient cells (~ 10^7^ cells) and 4 μl of donor cell plasmid DNA, was spotted onto a non-selective plate and then incubated for 24 h at 37 °C in an Anoxomat jar adjusted to contain the appropriate atmospheric conditions. After overnight incubation, the transformation mixture was harvested from the surface of the plate into 1 ml of PBS. Finally, 50 μl of this suspension were inoculated on LB (non-selective and selective) plates 8% FCS and 10 μg/ml kanamycin and the plates were incubated for 3 days. The transformation efficiency was determined by the number of CFU in selected plates divided by the total CFU in non-selective media. It should be noted that in each transformation experiment, the SS1 strain of *H. pylori* without added DNA was also tested on the selective medium (10 μg/ml kanamycin) as a negative control. In any case, no clones were seen. Also, at the beginning of each step of this process, we repeated the urease broth test to confirm the SS1 strain of *H. pylori*.

### Extraction and evaluation of plasmid DNA

The plasmid pattern of the recombinant strain was evaluated for confirming the transformation process. The Plasmid was extracted from fresh cultured strains using FavorPrep™ Plasmid Extraction Mini Kit (Pintung, Taiwan) according to the manufacturer’s instructions. Horizontal electrophoresis system (Bio-Rad USA) was used to visualization of plasmid pattern by agarose gel electrophoresis. In addition, plasmid DNA quality was measured by an ultraviolet spectrophotometer (Nano drop Technologies, Inc., Wilmington, DE, USA) at 260 and 280 nm.

### Plasmid DNA sequence analysis and PCR assay

For verification, plasmids were sequenced with T7-F promoter (5′ TAATACGACTCACTATAGGG 3′) and T3-R promoter (5′ ATTAACCCTCACTAAAGGGAA 3′) primers for pGPU6/GFP/Neo constructs. The reaction mixture for polymerase chain reaction (PCR) assay was 25 μL that was prepared as follows: 1X Taq premix Master mix (Ampliqon, Denmark), 0.4 pmol of each forward and reverse primer (Table 1), and 3 μl of purified plasmid DNA sample. The PCR temperature program was performed as follows: an initial denaturation step for 5 min at 95 °C, followed by 34 cycles at 95 °C for 30 s, 52 °C for 45 s, and 72 °C for 1 min, and final extension at 72 °C for 10 min in a Bio-Rad thermal cycler (Bio-Rad Laboratories, Inc., USA). The products PCR were subjected to 2% Agarose gel electrophoresis.

**Table 1:** qPCR primers for identification of *H. pylori ureB*, *ureC* and *cagA* genes

Gene	Primer sequence (5’ to 3’)		Product length (bp)	Reference
	**Forward**	**Reverse**		
***ureB***	CTCAATCACCAGTTCTGACTC	GATCGCTGGGTTAATGGTG	178	This study
***ureC***	GATAAGTTTGTGAGCGAATGC	TTGCTTTCTAACACTAACGC	146	This study
***cagA***	AGCGACCTTGAAAATTCCG	TTGAGATCGGCTAACGCTT	140	This study

### Sequencing

Sequencing was done by Bioneer Co., Korea mediated by Pishgam Co., Iran, and the data was analyzed using BioEdit software version 7.5.2 [31].

### Effect of urease inhibition in *H. pylori*

In order to investigate the inhibitory effect of siRNA on the urease activity of *H. pylori* recombinant strain, the activity of this enzyme was evaluated by urea broth medium (Difco, Detroit, MI, USA), compared to the non-recombinant strain. Urea broth medium was prepared as follows: 10 g of urea, 0.05 of yeast extract, 4.5 g of potassium phosphate, monobasic, 4.7 g of potassium phosphate, dibasic and 0.005 g of Phenol red. All ingredients were dissolved in 500 ml of distilled water and after filtering (pore size 0.45 mm). Then the pH was adjusted to 6.8 and 1 ml of the solution, added to the sterilized 1.5 ml microtubes and stored at 4 °C. A concentration of 1 McFarland was prepared from recombinant and non-recombinant SS1 strains of *H. pylori* and 100 μl of each strain was added to the urea broth medium and the result was read after incubation.

### RNA extraction and qPCR

Total RNA extraction was performed using the FavorPrep™ RNA extraction kit (Pintung, Taiwan) according to the manufacturer's instructions. Briefly, after overnight cultivation of *H. pylori SS1* recombinant strain, which was in microaerophilic conditions, a suspension was prepared and 200 μL of lysing buffer was added to it and incubated for 10 min at room temperature. Then 1 ml of YTzol was added to the suspension and centrifuged at 12,000 rpm for 3 min. By adding and repeatedly centrifuging the material, finally, the purified RNA was stored at -20 °C. The purity of the RNA preparations was also analyzed using an ultraviolet spectrophotometer (Nano drop Technologies, Inc., Wilmington, DE, USA). cDNA synthesis was performed with FavorPrep™ RNA extraction kit (Pintung, Taiwan) according to the manufacturer's instructions.

For quantitative PCR (qPCR) Each reaction mixture consisted of 5 μl of cDNA, 1 μl each of forward and reverse primers, 5 μl of nuclease-free water and 13 μl of SYBR Green PCR Master Mix in a total reaction volume of 25 μl (96-well optical plates). The thermal cycler program consisted of a preliminary step of 10 min at 95 °C; 45 cycles of 15 s at 95 °C; and 1 min at 60 °C using SYBR Green Master Mix (Applied Biosystems; Thermo Fisher Scientific, Inc.). qPCR assay was performed in duplicate and the presented results are the average of these assays and are expressed in copies per milliliter. The sequences of primers in this study were designed by Oligo 7 software (Molecular Biology Insights, Inc., Cascade, CO, USA) and OligoAnalyzer online server (https://www.idtdna.com/pages/tools/oligoanalyzer) and synthesized by Invitrogen (Shanghai, China). Also, primers were aligned by multiple sequence alignment (MSA) method using Clustal Omega software (https://www.ebi.ac.uk/Tools/msa/clustalo/). The sequence of gene expression primers is shown in Table 1.

### Statistical analysis

REST (Relative Expression Software Tool) software was used for statistical analysis of relative expression results in real-time PCR [32]. The obtained results were analyzed using paired t-test and P < 0.05 was considered significant.

## Results

### Transformation of *H. pylori* SS1 strain

**Fig. 4:**
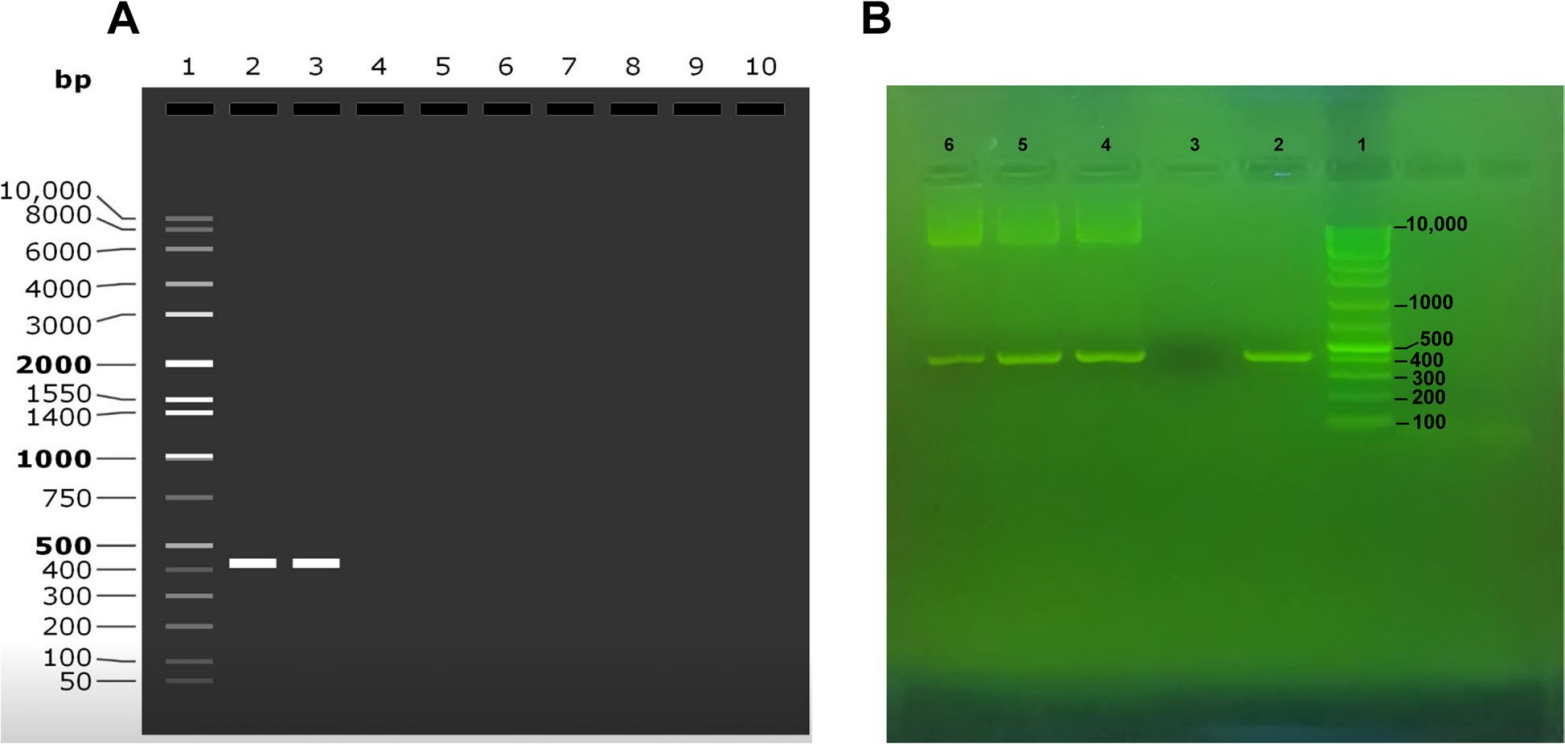
**A** In silico simulation for pGPU6/GFP/Neo plasmid constructs model using SnapGene software; Lane 1: 1 Kb DNA ladder; Lanes 2 and 3: 426 bp in silico digested product for the recombinant strain containing siRNA-ureB and siRNA-cagA using the in silico method. **B** Confirming the presence of the transformed pGPU6/GFP/Neo plasmid along with the inserted fragments; Lane 1: 1 Kb DNA ladder; Lanes 2, 4, 5 and 6: 426 bp product for the recombinant strain containing siRNA-UreB and siRNA-CagA; Lane 3: negative control

The number of colonies of transformed bacteria on LB agar medium was uncountable, which indicates the high efficiency of the natural transformation method. Plasmid profiling of several suspected colonies proved that the colonies contained pGPU6/GFP/Neo plasmid. PCR confirmed the presence of pGPU6/GFP/Neo plasmid as well as *ureB* and *cagA* genes (Fig. 4B).

### Sequence selection and evaluation of candidate siRNAs

Different siRNAs were obtained in the siDirect 2.0 web server, and finally siRNA-UreB-1352 5'- AAGGTGGGTTCATTGCATTAA-3' and siRNA-CagA-1656 5'- AGGCGGAATTTAGAGGATAAA-3' were selected for synthesis. It was found that the GC content for both siRNAs was 38%. Our findings showed that the binding energy for siRNA-UreB and siRNA-CagA was -28 and -25.2, respectively, with an inhibition efficiency of about 0.9, which allowed us to predict usefulness for in vitro evaluation.

### In silico cloning

**Fig. 5:**
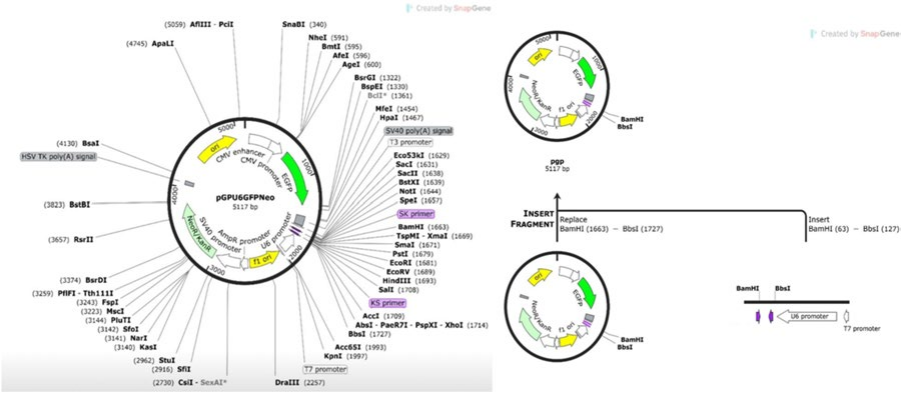
In silico cloning of the pGPU6/GFP/Neo expression vector designed using SnapGene software (version 5.2.3) (https://www.snapgene.com/)

BamHI (GGATCC) and Bbs I (GAAGAC) restriction sites were added to the upstream and downstream of the pGPU6/GFP/Neo plasmid constructs model sequence. Finally, we used SnapGene software to integrate the adapted DNA sequence to the pGPU6/GFP/Neo vector, between the BamHI and Bbs I restriction sites (Fig. 5). In silico digestion and gel electrophoresis simulation confirmed the desired final products, shown in Fig. 4A.

### In vitro effects of urease inhibition

**Fig. 6:**
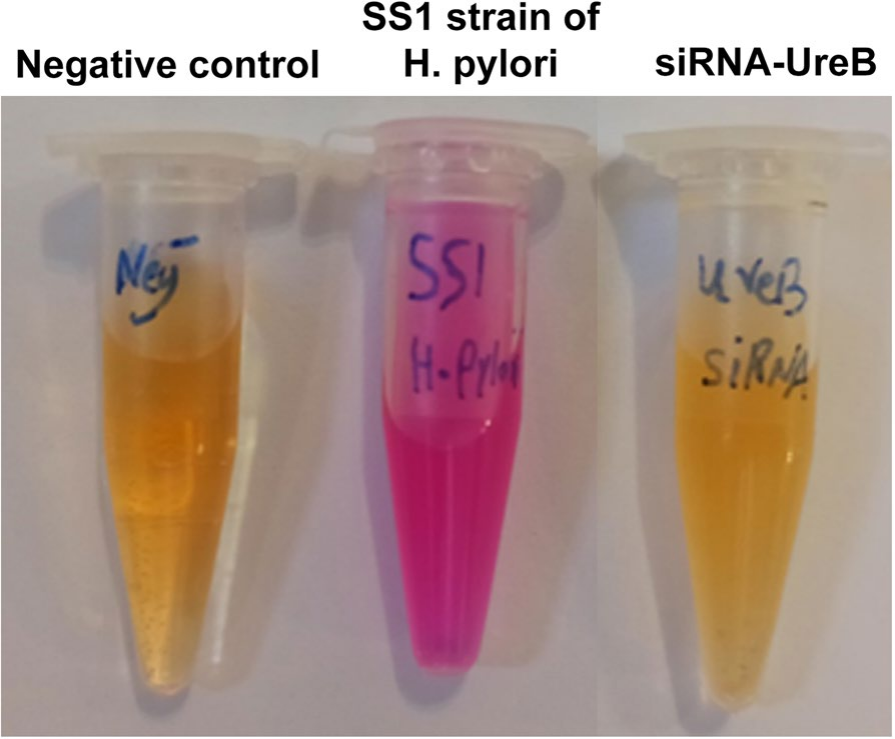
Inhibitory effect of urease activity

In this study, we investigated the urease inhibitory effect using urea broth medium (pH 6.8). The native strain of *H. pylori* SS1 was used as a positive control along with the recombinant strain by siRNA-UreB. Our results showed that siRNA-UreB could successfully prevent the urease enzyme activity of this bacterium in urea broth environment (Fig. 6).

### *ureB* and *cagA* genes expression in* H. pylori* SS1 strain after siRNA transfection

**Fig. 7:**
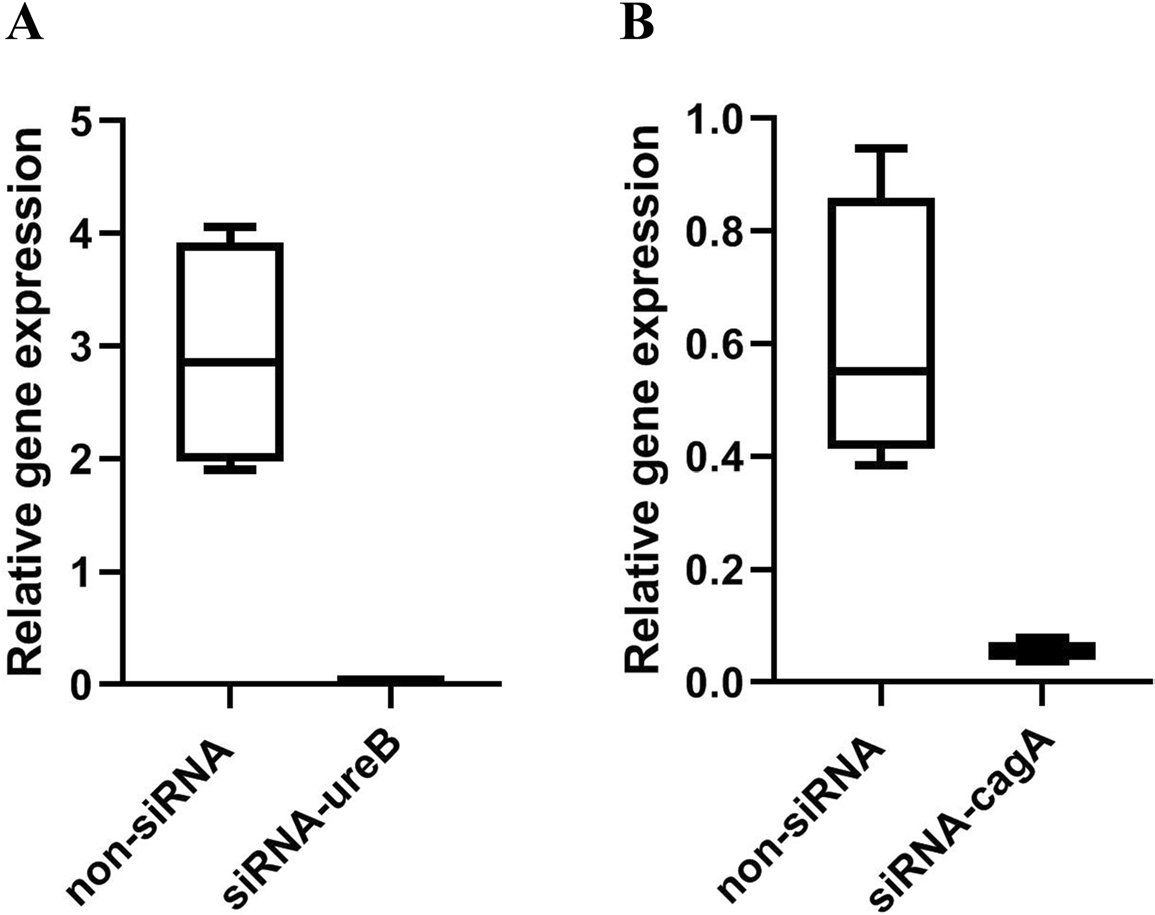
Box plots of qPCR data. **A** Boxplot of the expression level of siRNA-UreB in the recombinant strain SS1 decreased by about 5000 fold compared to the native strain of *H. pylori* SS1. **B** Boxplot of the expression level of siRNA-CagA in the recombinant strain SS1 decreased by about 1000 fold compared to the native strain of *H. pylori* SS1

We determined the expression level of *ureB* and *cagA* genes in *H. pylori* strain SS1 with and without siRNA transfection by real-time PCR method. The 2^−ΔΔCt^ method was used to determine the difference in normalized *ureB* and *cagA* gene expression caused by different siRNAs. Our results showed that siRNA-ureB and siRNA-cagA can significantly reduce level of gene expression in comparison with non-siRNA (P < 0.05). Meanwhile, the expression levels of siRNA-UreB and siRNA-CagA in the recombinant strain SS1 were reduced by about 5000 and 1000 fold, respectively, compared to the native *H. pylori* strain SS1 (Fig. 7).

## Discussion

*H. pylori* is strongly interested in unique colonization in the deep mucosal layer of the stomach. Several mechanisms including motility, urease production, adhesion, and CagA are important in *H. pylori* colonization [2]. This pathogen uses its urease activity to neutralize the acidic conditions of the host's stomach. Specific interactions between bacterial adhesins and host cell receptors lead to successful colonization and persistent infection. Finally, the bacterium releases several virulence factors (such as CagA, urease, VacA) that cause host tissue damages [9]. In this regard, several studies have been conducted to reduce colonization by inhibiting virulence factors.

*H. pylori* is a natural competence along with, *N. gonorrhoeae, S. pneumoniae* and *B. subtilis*. In this bacterium, natural competence is associated with proteins in the comB locus, a feature that is not found in other bacteria with natural competence [30, 33, 34]. A pGPU6/GFP/Neo siRNA construct containing siRNA inhibiting *ureB* or *cagA* was designed and synthesized. We used the natural competence method to transform the constructs into *H. pylori* SS1 strain in order to inhibit *ureB* or *cagA* pathogenicity factors, and their inhibition effect were analyzed phenotypic and genotypically.

Urease is a virulence factor associated with pathogenicity in various pathogenic bacteria, that is essential in the colonization and maintenance of bacterial cells in host tissues [35, 36]. In several studies, the inhibition of *H. pylori* urease enzyme by chemical compounds has been shown. Unfortunately, the investigations that has been done so far could not provide the desired effects to inhibit this enzyme, which include hydrolytic instability, toxicity, and adverse side effects [37]. One of these compounds is fluorofamide (N-(diaminophosphinyl)-4-fluorobenzenamide), which cannot inhibit urease due to its instability in acidic conditions [38]. In two studies possible in vitro and in vivo inhibitory effect of Palmatine (Pal) from *Coptis chinensis* on urease were evaluated. The results showed that Pal, which targets sulfhydryl groups, emerged as a promising candidate as a urease inhibitor [12, 39]. In addition, Macegoniuk et al. used a group of urease inhibitors, namely aminophosphinic acid and aminophosphonic acid derivatives, against *H. pylori* urease in laboratory conditions. The results of this study showed that bis (N-methylaminomethyl) phosphinic acid acted as the most effective inhibitor in the sensitivity profile studies of *H. pylori* J99 [40]. Another compound that has been evaluated to inhibit urease enzyme activity is Acetohydroxamic acid (AHA). AHA is structurally similar to urea and has an effective activity in inhibiting the bacterial urease enzyme, but some limitations related to severe side effects, such as psycho-neural and muscular-other symptoms, have led to the limited use of this treatment [41, 42].

So far, no study has been conducted to inhibit the urease enzyme activity from *H. pylori* using siRNA. In this study, we evaluated the effect of siRNA to inhibit urease enzyme activity using two phenotypic and genotypic methods in *H. pylori* SS1 strain for the first time. After examining the basic characteristics of siRNA and confirming the inhibitory effect of this molecule, we can conclude that in the future siRNA-UreB can have a potential effect in helping the treatment.

CagA is able to activate NF-κB and translocate it to the nucleus, where it regulates the transcription of IL-8, a chemotactic and inflammatory cytokine. To further confirm the role of NF-κB, Yang et al. transfected NF-κB-specific siRNA into gastric cancer cells and succeeded in downregulating NF-κB expression [43]. Also, studies showed that the use of siRNA against CagA mRNA as an inhibitor led to a decrease in IL-8 production at different times (6, 12 and 24 h after electroporation) [44]. In the other hand, CagA negatively regulates autophagy and promotes inflammation in *H. pylori* infection, which is regulated by the activation of the c-Met-PI3K/Akt-mTOR signaling pathway. Li et al. showed that c-Met siRNA significantly affected CagA-mediated autophagy and decreased the levels of p-Akt, p-mTOR, and p-S6 [45]. Signal transducer and activator of transcription 3 (STAT3) is a transcription factor encoded by the STAT3 gene in humans. To investigate the role of STAT3 in mediating CagA-dependent regenerating islet-derived protein 3 gamma (REG3γ) transcription, a STAT3-specific siRNA was used. The results showed that transfection of a STAT3-specific siRNA led to almost complete depletion of the total STAT3 protein [46]. In addition, Barry and colleagues investigated the effect of a new inhibitor, D,l-a-difluoromethylornithine (DFMO), in *H. pylori*-infected mice. The findings of their study showed that DFMO inhibited the expression level of *H. pylori cagA* gene and its phosphorylation in gastric epithelial cells, which was associated with the reduction of interleukin-8 expression [47]. We used the pGPU6/GFP/Neo plasmid to transfer the *cagA* gene, and finally the qPCR results showed a decrease in the expression level of this gene up to 1000 fold. These findings indicated that siRNA-CagA may contribute to the effectiveness of reducing gastritis and colonization.

In general, based on the results of this study, it was found that siRNA can have a potential effect in reducing or inhibiting the activity of *ureB* and *cagA* and reducing the pathogenicity of *H. pylori* and act as a promising perspective to suppress other pathogenic factors of this pathogen.

## Conclusion

It conclusion siRNA-ureB and siRNA-cagA were reduced the expression level of UreB and CagA, and as a result, *H. pylori* colonization and inflammation in the gastric mucosa were decreased. Finally, the results of this study showed that siRNA can be promising for the treatment of *H. pylori* infection, but more in vivo studies are needed regarding the effectiveness of siRNA on diseases associated with this pathogen.

### Limitations

The main limitations of this study is the lack of an animal model to investigate pGPU6/GFP/Neo plasmid recombinant clinical isolates.

## Data Availability

All the data supporting the findings are contained within the manuscript.
